# Traditional Chinese Medicine Protects against Cytokine Production as the Potential Immunosuppressive Agents in Atherosclerosis

**DOI:** 10.1155/2017/7424307

**Published:** 2017-09-06

**Authors:** Yan Ren, Wei Qiao, Dongliang Fu, Zhiwei Han, Wei Liu, Weijie Ye, Zunjing Liu

**Affiliations:** ^1^Department of Cardiology, China-Japan Friendship Hospital, Beijing 100029, China; ^2^Department of Neurology, China-Japan Friendship Hospital, Beijing 100029, China

## Abstract

Atherosclerosis is a chronic inflammatory disease caused by dyslipidemia and mediated by both innate and adaptive immune responses. Inflammation is a critical factor at all stages of atherosclerosis progression. Proinflammatory cytokines accelerate atherosclerosis progression, while anti-inflammatory cytokines ameliorate the disease. Accordingly, strategies to inhibit immune activation and impede immune responses towards anti-inflammatory activity are an alternative therapeutic strategy to conventional chemotherapy on cardiocerebrovascular outcomes. Since a number of Chinese medicinal plants have been used traditionally to prevent and treat atherosclerosis, it is reasonable to assume that the plants used for such disease may suppress the immune responses and the resultant inflammation. This review focuses on plants that have immunomodulatory effects on the production of inflammatory cytokine burst and are used in Chinese traditional medicine for the prevention and therapy of atherosclerosis.

## 1. Introduction

Atherosclerosis (AS), a chronic inflammatory disease characterized by dyslipidemia, is the most common type of cardiocerebrovascular disease and the leading cause of morbidity and mortality in the world nowadays [1, 2]. The characterization of atherosclerosis as an immune-mediated aberrance is based on evidence of immune activation and inflammatory signaling in human atherosclerotic lesions [3], the significance of inflammatory biomarkers as independent risk factors for cardiocerebrovascular events [4], as well as the ability of LDL particles and their contents to activate innate and adaptive immunity [5, 6]. Thus, strategies to inhibit immune activation and impede immune responses towards anti-inflammatory activity are an alternative therapeutic strategy to conventional chemotherapy on cardiocerebrovascular outcomes. In accordance with ancient Chinese pharmacopoeias, a large amount of medicinal plants has shown inhibitory potentials in immune responses and some of them have been used traditionally to prevent and treat atherosclerosis. This is partly due to their safety and lower side effects and, in some ways, their more effectiveness. As inflammation is a critical factor at all stages of atherosclerosis progression, from attracting immune cells and atherosclerotic plaque formation to its rupture, cytokines are major mediators in all kinds of inflammation [7, 8]. In this review, we focus on plants that are used in traditional Chinese medicine (TCM) and have been reported to act as immunomodulatory agents of suppressive function on cytokine production in atherogenesis.

## 2. Role of Inflammatory Cytokines in the Pathogenesis of Atherosclerosis

The concept of atherosclerosis as an inflammatory disease is based on the evidence that inflammatory cells are abundant in atherosclerotic lesions, which are the major source of cytokine that was involved in all stages of atherosclerosis and have a profound impact on the pathogenesis of this disease [9]. Cytokines are protein mediators produced by monocytes, macrophages, T cells, and platelets, as well as by ECs, smooth muscle cells (SMCs), and adipocytes, in answering to inflammation and other stimuli [10, 11].

Recently, emphasis has been placed on the role of cytokines and the way they act on a variety of objects exerting multiple effects and are largely responsible for the crosstalk among endothelial, smooth muscle cells, leukocytes, and other vascular residing cells having a potentially causative role in atherosclerosis. For example, cytokine-induced activation of ECs can induce endothelium dysfunction accompanied by upregulation of adhesion molecules and chemokines, such as intercellular adhesion molecule-1 (ICAM-1), vascular cell adhesion molecule-1 (VCAM-1), and monocyte chemoattractant protein-1 (MCP-1), which promotes migration of immune cells (monocytes, neutrophils, and lymphocytes) into the atherosclerosis lesion [12, 13]. Moreover, cytokines impress the function of SMCs by promoting their growth, proliferation, and migration. Studies by Cushing et al. demonstrated that minimally ox-LDLs, but not native LDLs, give rise to MCP-1 production in vascular wall cells such as endothelial cells and smooth muscle cells and MCP-1 functions in the recruitment of monocytes to atheroma [14]. Finally, at later stages of atherosclerosis, proinflammatory cytokines accelerate destabilization of atherosclerotic plaques, apoptosis of various cells, and matrixed gradation, thereby accelerating plaque breakage and thrombus formation. For instance, TNF-*α* and IL-1*β* accelerate apoptosis of macrophages together with foam cells leading to the enlargement of the lipid core [15, 16]. Such cytokines also irritate apoptosis of SMCs leading to thinning of the fibrous cap. Further remodeling of the extracellular matrix (ECM) is controlled by a series of proteases, particularly matrix metalloproteinases (MMPs), and their inhibitors (tissue inhibitor of metalloproteinases (TIMPs)) produced by macrophages and other vascular cells [17]. The expression and/or activities of MMPs and TIMPs are adjusted by cytokines [18]. Vulnerable plaques have very few SMCs and high macrophage substance and are susceptible to rupture leading to thrombosis in the end [19]. What is more, key components involved in thrombosis are also the target of regulation by cytokines [20].

To date, atherosclerosis cannot be reversed by medical treatment, warranting the need for better understanding of this pathology in order to develop new strategies to struggle this deadly disease. Targeted intervention strategies on reducing proinflammatory cytokine expression may be of great help to improve current cardiovascular outcomes.

## 3. Immunosuppressive Role of Chinese Medicinal Plants on Inflammatory Cytokines Expression in Atherogenesis

Experimental endeavors to control atherosclerosis have included both wide-spectrum anti-inflammatory and immunomodulatory approaches, as well as specific targeting of mechanisms [21, 22]. Given traditional Chinese medicine is deeply rooted in the history and has been widely used to prevent and treat cardiocerebrovascular disease; it is reasonable to assume that these plants used for such diseases may refrain the immune responses and the resultant inflammation. Furthermore, trying to describe the potential clinical predictive value of some TCMs on proinflammatory cytokine expression in the progression and complications of atherosclerosis may be of great help not only in understanding TCMs but also in determining the potential of cytokine-based therapies. In consideration of the variety of cells that participate in atherogenesis, the large number of cytokines that is expressed by each of them and the pleiotropic activity of each cytokine; it is almost impossible to describe minutely all interactions taking place during atherogenesis [23]. Herein, ICAM-1, VCAM-1, MCP-1, TNF-*α*, IL-1*β*, and MMPs-9, which participate in initial and later stages of atherosclerosis [24], will be more elaborately discussed.

### 3.1. Intercellular Adhesion Molecule (ICAM-1) and Vascular Cell Adhesion Molecule (VCAM-1)

Inflammatory stimuli result in the upregulation of adhesion molecules, which is a critical feature in early atherosclerosis. ICAM-1 and VCAM-1 are the main adhesion molecules which are important for the firm adhesion of leukocytes to the endothelium. These adhesion molecules in turn enable the adhesion of mononuclear leukocytes to endothelial cells and also their transmigration into the intima, further leading to a series of inflammatory reactions, which finally aggravates plaque instability [25, 26]. Accordingly, inhibiting monocyte adhesion to the endothelium is considered a novel treatment strategy for atherosclerosis.

*Eucommia ulmoides* Oliver is the only known species of the genus Eucommia. Pharmacologically, researchers reported that long-term Eucommia leaf extract (ELE) intake can effectively improve vascular function by promoting plasma nitric oxide (NO) levels while suppressing the production of ICAM-1 and VCAM-1 [27]. Likewise, Hosoo et al. examined the effects of ELE administration on artery function and morphology in spontaneously hypertensive rats (SHRs) and found that ELE significantly perfected Ach-induced aortic endothelium-dependent relaxation as compared to animals taking a normal diet. Plasma NO levels and media thickness were significantly increased and decreased, respectively, in the ELE-treated SHRs, indicating that ELE may exert anti-endothelial dysfunction and antioxidant and antiatherogenic effects [28].

*Polygonum multiflorum* stilbene glycoside (PMS) is a water-soluble fraction of *Polygonum multiflorum* Thunb., one of the most famous tonic traditional Chinese medicines, that has protective effects on the cardiocerebrovascular system [29]. Yang et al. studied the function of PMS on macrophage-derived foam cell functions and found that PMS could reduce the high production of intercellular adhesion molecule- (ICAM-) 1 protein and the vascular endothelial growth factor (VEGF) protein levels in the medium induced by oxidized lipoprotein when analyzed by flow cytometry, suggesting that PMS is a powerful agent against atherosclerosis and that PMS action could possibly be through the inhibition of the production of ICAM-1 and VEGF in foam cells [30].

Genistein, a major isoflavone in soy and red clover, has won wide attention due to its potential beneficial impacts and various biological actions [31]. Recent human intervention researches using soy phytoestrogens proved their beneficial effect on atherosclerosis. For instance, studies by Jia et al. demonstrated that genistein at physiological concentrations (0.1 *μ*M–5 *μ*M) significantly restrained TNF-*α*-induced expression of adhesion molecules and chemokines such as ICAM-1 and VCAM-1, which play a critical role in the firm adhesion of monocytes to activated endothelial cells [32].

In light of the established pharmacological role of muscone, which is a pharmacologically active component isolated from musk, Wu et al. reported that the administration of muscone may reduce cardiac remodeling and improve cardiac function following MI, indicating a beneficial effect on the protection against the development of atherosclerotic lesions [33].

### 3.2. Monocyte Chemoattractant Protein-1 (MCP-1)

MCP-1 and its receptor play key roles in monocyte recruitment during foam cell and fatty streak formation in atherogenesis. Studies prove that circulating blood monocytes are the precursors of foam cells that are made up of lipid-laden macrophages. Overexpression of MCP-1 in specific tissues induces a localized infiltration of monocyte/macrophages. Endothelial expression of MCP-1 is thought to cause the subendothelial migration of monocytes in early atherosclerotic lesions. Upon stimulation, macrophages are able to produce significant loss of MCP-1 in atherosclerotic lesions. Recent researches indicate that homocysteine stimulates MCP-1 expression in cultured endothelial cells, giving rise to enhanced monocyte adhesion to endothelial cells as well as increased chemotaxis. In addition to recruiting and accumulating monocyte into the inflammatory sites, such as atherosclerotic lesions, MCP-1 also mediates the development of medial thickening [34, 35].

Wogonin (Wog) is an active component isolated from *Scutellaria baicalensis* radix, possessing antioxidant and anti-inflammatory properties [36]. Chang et al. found the effect of Wog on phorbol myristate acetate- (PMA-) induced MCP-1 expression in human umbilical vein endothelial cells (HUVECs) and measured the MCP-1 mRNA levels and MCP-1 release in Wog-treated HUVECs and reported that Wog inhibits MCP-1 induction in HUVECs. Furthermore, this inhibition is mediated by suppressing AP-1 transcriptional activity via the attenuation of extracellular signal-regulated kinase1/2 (ERK1/2) and c-Jun N-terminal kinase (JNK) signal transduction pathways, indicating that Wog has the potential therapeutic advantage for use in anti-inflammatory and vascular disorders [37].

Tetramethylpyrazine (TMP), a pharmacologically active component isolated from the rhizome of the Chinese herb Rhizoma Chuanxiong (Chuanxiong), has been clinically used in China and Southeast Asian countries for the prevention and treatment of cardiocerebrovascular diseases for about fifty years [38]. Recently, the function of TMP on the critical components of atherogenesis has been intensively investigated. Wang et al. reported that TMP reduces MCP-1 levels in the plasma and inhibits lectin-like oxidized LDL receptor-1 (LOX-1) production in rabbit aortas. Likewise, in their in vitro study, they revealed that TMP inhibits the ox-LDL-induced activation of p-ERK, p-p38, and p-JNK mitogen-activated protein kinase (MAPK), proving that TMP protects the endothelium and prevents atherosclerosis via inhibition of immunological responses [39].

Curcumin, a major active component in turmeric, has anti-inflammatory, anti-oxidative, and anti-atherosclerotic properties [40]. Liu et al. studied the effect of curcumin on ox-LDL-induced MCP-1 production and cholesterol efflux in macrophages and revealed that curcumin significantly suppressed the MCP-1 production induced by ox-LDL and enhanced cholesterol efflux through the inhibition of JNK pathways, which suggest that the vascular protective effect of Curcumin is related to anti-inflammation and antiatherosclerosis [41].

Berberine is the main component of the traditional Chinese medicine umbellatine, which has a widespread property and was used to treat many diseases clinically [42]. Chen et al. investigated the function and the mechanism of action of berberine on the production and secretion of MCP-1 in vitro to identify new pharmacological actions of it and found that berberine may suppress the expression and secretion of the MCP-1 in macrophages stimulated by acetylated low-density lipoprotein (AcLDL), whereas the peroxisome proliferator-activated receptor (PPAR*γ*) inhibitor could recede this effect of berberine, which reveals that berberine may inhibit the production of MCP-1 in AcLDL-stimulated macrophages and, at least in part, be regulated through activation of PPAR*γ* [43].

### 3.3. Tumor Necrosis Factor-*α* (TNF-*α*)

TNF-*α* is a proinflammatory molecule expressed by cellular components of early fatty streaks and late atherosclerotic lesions of humans. Moreover, TNF-*α* may be secreted by several cell types within atherosclerotic plaques, such as endothelial cells, SMCs, and macrophages. However, monocytes and macrophages are the main sources of TNF. Researches indicate that circulating levels of TNF-*α* are increased in advanced atherosclerosis and in patients symptomatic for acute stroke. In mice, TNF-*α* gene knockout on the ApoE−/− background results in significantly smaller atherosclerotic plaque areas [44, 45]. Together, these data indicate that TNF-*α* may represent a promising target to reduce atherosclerosis.

Paeonol is the main active component of Cortex Moutan, which has several effects in traditional Chinese medicine, possessing various pharmacological activities, particularly an antiatherosclerosis effect [46]. Li et al. studied the association of the therapeutic effect of paeonol on atherosclerotic rabbits with its anti-inflammatory function, and their histological analysis of rabbits on the high-fat diet with paeonol showed significant improvement in atherosclerosis plaque. Moreover, the blood levels of TNF-*α* and the translocation of NF-*κ*B to the nucleus were significantly inhibited in paeonol groups, as was the suppression of lipid peroxidation, which indicates that the anti-inflammatory function of paeonol may contribute to its antiatherosclerosis effect [47].

Tanshinone IIA (Tan IIA) as a major component to exert therapeutic effects of danshen is a traditional Chinese medicine commonly used in Asia for the prevention and treatment of cardiocerebrovascular diseases, such as atherosclerosis [48]. Wang et al. investigated the putative protective function of Tan IIA on endothelial progenitor cells (EPCs) injured by tumor necrosis factor-*α* (TNF-*α*) and showed that TNF-*α* impaired EPC proliferation, migration, adhesion capacity, and vasculogenesis ability in vitro as well as promoted EPC secretion of inflammatory cytokines. However, Tan IIA was able to reverse these effects, which revealed that Tan IIA may have the potential to protect EPCs against damage induced by TNF-*α*, offering evidence for the pharmacological basis of Tan IIA and its potential use in the prevention and treatment of early atherosclerosis associated with EPC and endothelial damage [49].

Catalpol, isolated from the roots of *Rehmannia glutinosa*, Chinese foxglove, is an iridoid glycoside with antioxidant, anti-inflammatory, and antihyperglycemic agent [50]. Liu et al. investigated the function of catalpol on diabetic atherosclerosis in alloxan-induced diabetic rabbits and showed that catalpol treatment ameliorated diabetic atherosclerosis in diabetic rabbits as revealed by significantly suppressed neointimal hyperplasia and macrophage recruitment. Catalpol treatment also increased the activities of superoxide dismutase and glutathione peroxidase and enhanced the plasma levels of total antioxidant status, meanwhile decreasing the levels of malondialdehyde, protein carbonyl groups, and advanced glycation end product. What is more, catalpol also reduced circulating levels of TNF-*α*. In a word, these data collectively indicated supporting evidence and important novel pharmacological function of catalpol, which may have potential therapeutic value for the treatment and/or prevention of atherosclerosis in diabetic patients [51].

### 3.4. Interleukin-1*β* (IL-1*β*)

IL-1*β*, as a gatekeeper of inflammation, is a proinflammatory cytokine produced by myeloid cells. Secretion of IL-1*β* cytokine and expression of their receptor are enhanced in atherosclerotic aortas. IL-1*β* is an essential factor of Th17 cell differentiation that can facilitate inflammation in the vascular wall. Experiments in mouse models confirmed the proatherogenic nature of IL-1*β* that is involved in the upregulation of adhesion molecule production by endothelial cells as well as macrophage activation. Therefore, strategies for the prevention of IL-1*β* burst resolves inflammation in consideration of how the cytokine is released from the cell or how the precursor is cleaved [52, 53].

*Toona sinensis* is well known as a traditional Chinese medicine; Yang et al. evaluated the protective function of noncytotoxic concentrations of aqueous leaf extracts of *Toona sinensis* (TS extracts) in human umbilical vein endothelial cells (HUVECs) and showed that HUVECs were preincubated with TS extracts which resulted in enhanced resistance to oxidative stress and cell viability in a dose-dependent manner. In addition, IL-1 *β* was positively correlated with cytotoxicity and negatively with TS extract concentrations. Notably, TS extract treatment significantly restrained reactive oxygen species (ROS) generation in HUVECs, which supported the traditional use of *Toona sinensis* in the treatment of free radical-related diseases and atherosclerosis [54].

Fistular onion stalk, derived from *A. fistulosum*, is used as a traditional herbal medicine, and its extract exhibits certain beneficial advantages on cardiocerebrovascular disorders [55]. He et al. examined the function of fistular onion stalk extract on the pathological features, circulating inflammatory cytokines in in vivo model of atherosclerosis, and reported that rats treated with fistular onion stalk extract showed a significant decrease in the pathological region compared with the vehicle-treated controls. Furthermore, the extract also restrained the levels of the local inflammatory cytokines IL-1*β* and IL-6, suggesting that fistular onion stalk extract may be helpful for the attenuation of atherosclerosis [56].

*Andrographis paniculata* (Burm.f.) Nees (*Acanthaceae*) is a long-established therapeutic herb. Recently, epidemiologic evidence has revealed significant associations between atherosclerosis and *Porphyromonas gingivalis* (Pg) [57]. Al Batran et al. examined the function of andrographolide (AND) on atherosclerosis induced by Pg in rabbits and found that rabbits treated with AND showed significant reduction in TC, TG, and LDL levels and significant promotion in HDL level in the serum. Moreover, the treated rabbits showed reductions in interleukins (IL-1*β* and IL-6) as compared to the atherogenic group, which could be attributed to the anti-inflammatory effect of AND, which was involved in the decrease of proinflammatory cytokines [58].

Scropolioside B isolated from *Scrophularia dentata* Royle ex Benth is used for antiviral and anti-inflammatory treatment [59]. Zhu et al. investigated whether scropolioside B exhibits anti-inflammatory function and further analyzed its underlying mechanism in human monocytes and showed that scropolioside B significantly diminished the production and secretion of IL-1*β* and IL-32; furthermore, their study also revealed that this is regulated by modulating NF-*κ*B levels, which strengthen the previous notion of the anti-inflammatory effects of iridoids and highlight scropolioside B as a potential herb for the treatment of rheumatoid arthritis and atherosclerotic disease [60].

### 3.5. Matrix Metalloproteinases (MMPs)

Atherosclerotic plaque rupture causes most myocardial infarctions. MMPs are involved in the development and the progression of atherosclerosis and are related to an enhanced factor of cardiocerebrovascular morbidity and mortality: an increased MMP expression has been detected in atherosclerotic plaques, and their activity may be responsible for plaque instability and rupture and for a strengthen platelet aggregation. MMPs, also named matrixins, are subdivided into at least five groups based on their structure and/or substrate specificities. MMP family members include collagenases (MMP-1, MMP-8, MMP-13, and MMP-18), gelatinases (MMP-2 and MMP-9), stromelysins (MMP-3, MMP-10, and MMP-11), matrilysins (MMP-7 and MMP-26), and membrane-type MMPs (MMP-14 and MMP-15). It has been demonstrated that specific matrix metalloproteinases (MMPs) such as MMP-1, MMP-2, MMP-3, MMP-9, and MMP-14 have been proven to enhance angiogenesis, and in particular, an increase in MMP-9 plasma levels is related with higher all-cause mortality and cardiocerebrovascular mortality. Given MMPs have pleiotropic actions in atherosclerosis, strategy on a specific adverse effect of MMPs while leaving intact essential physiological functions, additionally, avoiding a narrow or nonexistent therapeutic window [61].

Red yeast rice (RYR) is a traditional Chinese medicinal agent prepared by using *Monascus purpureus* fermented with rice, which has been recorded in ancient Chinese pharmacopoeias since the Ming dynasty [62]. Red yeast rice extracts contain a mixture of starch, phytocholesterols, isoflavones, monounsaturated fatty acids, and polyketides called monacolins. Xie et al. used apolipoprotein E-deficient (ApoE−/−) male mice infused with angiotensin II to promote the development of atherosclerosis and showed that RYR extract significantly decreased atherosclerotic lesion areas in both the intima of aortic arches and cross sections of aortic roots. These functions were associated with reductions of serum total cholesterol, MMP-2, suggesting the potential interpretation that this traditional Chinese food herb may be used as a preventive treatment of atherosclerosis [63].

Alisma decoction (AD) is a classical traditional Chinese formula that was first prescribed in the Eastern Han dynasty, which composed of a combination of two herbs, including Alisma and Atractylodes [64]. Xue et al. examined the regulation of lipids and the anti-inflammatory function exerted by AD and evaluated the underlying molecular mechanisms using ox-LDL-stimulated foam cells derived from rat peritoneal macrophages and showed that AD markedly alleviated lipid deposition in foam cells as it inhibited the ox-LDL-induced expression of MMP-9. Collectively, their findings indicate that blocking lipid deposition and inhibiting inflammatory response may be one of the key mechanisms through which AD exerts its antiatherosclerotic effects [64].

Puerarin, a phytoestrogen derived from the Chinese medicinal herb radix puerariae, has been proven practical in the management of various cardiocerebrovascular diseases [65]. Li et al. examined the clinical significance of MMP-9 secreted by cultured monocyte-derived macrophages (HMDM) from patients with coronary heart disease (CHD) in vitro and evaluated the intervenient function of puerarin on them and revealed that the levels of MMP-9 secreted in vitro by HMDM from CHD patients could be used as indexes for evaluating patient's condition of ACS. In addition, puerarin can restrain the production and the activity of MMP-9 secreted by HMDM, stabilize the plaque, and perfect the vulnerability of blood to a certain extent [66].

Artemisinin, derived from the sweet wormwood *Artemisia annua*, has been used in the treatment of malaria in China for over 2000 years. Recently, artemisinin and its derivatives have been proven to have pharmacological actions beyond their antimalarial effects; these other properties include immunosuppressive and anti-inflammatory properties [67]. Wang et al. examined whether artemisinin could reduce MMP-9 production in phorbol myristate acetate- (PMA-) induced macrophages by regulating the protein kinase (PK) Cd⁄JNK⁄p38⁄ERK pathway and revealed that artemisinin significantly inhibited the induction of MMP-9 at both the transcriptional and translational levels in a dose-dependent manner in PMA-induced macrophages. In addition, artemisinin strongly blocked PKCd⁄JNK⁄p38⁄ERK MAPK phosphorylation, indicating that artemisinin may have a potential for use in the protection against the development of atherosclerotic lesions [68].

## 4. Author's Comments: Challenges in Exerting Advantages of Chinese Herb in Antiatherosclerosis

The immunopathology of atherosclerosis emphasizes the active inflammatory, complex or multifactorial, and long-term property of the disease. Understanding the characteristic and interrelationship in atherosclerosis may offer new concepts with possible impact not only on early and accurate diagnosis but also on preventive programs and perhaps more effective therapeutic interventions. In accordance with ancient Chinese pharmacopoeias, a large amount of medicinal plants have shown inhibitory potentials in antiatherosclerosis from the formation of fatty streak to plaque complications, which have been widely used to prevent and treat atherosclerosis in China. Moreover, the evidence-based study of TCMs in treating atherosclerosis suggested that, in addition to anticytokine production, Chinese herb also exerts anti-endothelial dysfunction and antioxidant and antihyperlipidemic effects, thus having widespread properties.

Recently, the Nobel Prize-winning discoveries of Professor Tu related to artemisinin from the Chinese herb *Artemisia carvifolia* won global interest in TCM. The Chinese natural herbs outlined in this review are potential targets for an immunomodulatory cardiocerebrovascular prevention strategy mainly due to its anti-inflammatory property. Despite encouraging results from either clinical trials or experimental, researches show that TCMs and their components or derivatives have immunomodulatory effects and supplied the rationale for the therapeutic potential of targeting inflammation in atherosclerosis; data from randomized, controlled, and double-blind clinical trials to evaluate cardiocerebrovascular outcomes for specific monomer from these natural herbs are a few and far between. Thus, future researches are required to explore detailed immunomodulatory molecular mechanisms of these medicinal herbs to elucidate the precise function of active ingredients and perfect their therapeutic management in atherosclerosis, and further large randomized controlled trials (RCTs) will shed light on the guidelines to define the appropriate target population, treatment periods, outcomes, and prediction of potential adverse effects.

## 5. Conclusions

The nature of atherosclerosis is chronic inflammation in the aortic, caused by dyslipidemia, innate and adaptive immune responses. Cytokines are elucidated to play a critical role in the interrelationship of atherosclerosis, such as in the initiation, progression, and even regression of atherosclerotic lesions. Given pro-nflammatory cytokines accelerate atherosclerosis progression, and anti-inflammatory cytokines ameliorate the disease; the balance between pro- and anti-inflammatory cytokines is the major element that determines the stability of atherosclerotic plaque [69, 70]. The Chinese natural herbs outlined in this review possess a widespread property beyond anti-endothelial dysfunction, antioxidant and antihyperlipidemic property; anti-inflammatory property is the most important mechanism partly in the prevention and treatment of atherosclerosis. However, precise immunomodulatory molecular mechanisms of these medicinal herbs and their active ingredients or derivatives in the therapy of antiatherosclerosis are a few and far between. Thus, there is still a long but promising journey between these Chinese medicinal herbs and their appropriate therapeutic management in atherosclerosis.
